# Vulvar Metastasis from Bladder Cancer

**DOI:** 10.1155/2015/324634

**Published:** 2015-04-27

**Authors:** Fouad Aoun, Elie El Rassy, Hampig Raphael Kourie, Eric Hawaux, Roland van Velthoven

**Affiliations:** ^1^Department of Urology, Jules Bordet Institute, Free University of Brussels (ULB), 1 Héger Bordet Street, 1000 Brussels, Belgium; ^2^Department of Oncology, Jules Bordet Institute, Free University of Brussels (ULB), 1 Héger Bordet Street, Brussels, Belgium

## Abstract

Vulvar metastasis of urothelial carcinoma of the bladder is a very rare entity; few cases are reported in the English literature. In this paper, we describe the clinical and pathological characteristics, evolution, and treatment of a patient with vulvar metastasis of urothelial carcinoma of the bladder followed by a brief review of the reported cases in the literature.

## 1. Introduction

Cancer of the vulva, primary or metastatic, is a very rare tumor constituting <1% of all female cancers. Particularly, primary vulvar cancers are responsible for 3–5% of gynecologic tumors whilst metastatic vulvar cancers are less common constituting 5–8% of vulvar cancers [1]. Metastases from bladder cancer most frequently involve the lymph nodes, bones, lung, liver, and peritoneum. Metastatic tumors sparing these five sites occur in 3% of the cases and affect the pleura, soft tissue, adrenal glands, or brain [2]. Metastasis to the female reproductive tract is rare. When it does occur, the genitourinary tract is the next most frequently reported primary origin [3]. Here, we report a vulvar metastasis of urothelial bladder carcinoma and describe its clinical and pathological features.

## 2. Case History

A 63-year-old multigravida, alcoholic, and heavy smoker female patient presented to our department late 2011 for stage IV small cell lung cancer. The diagnosis was established following surgical resection of a symptomatic metastatic cerebellar mass herniation. The patient received total brain radiotherapy and six cycles of Cisplatin-VP16 chemotherapy and was advised close follow-up.

The patient presented back in November 2012 with an abrupt onset of urinary incontinence. Investigations revealed stable lung disease and right side uretrohydronephrosis attributed to a bladder tumor. Transurethral resection of the tumor revealed a completely necrotic mass determined as urothelial carcinoma staged G3pT2. Consequently, we performed anterior pelvectomy that demonstrated a transitional cell carcinoma staged pT3bN0M0 and considered adjuvant treatment that the patient strictly refused.

On follow-up six months later, the patient was cachectic with a performance status of 4. Clinical exam revealed vaginal secretions, solid and tender right inguinal lymph nodes, and tumefaction of the right greater vulvae. Her thoracic and brain lesions were stable but retroperitoneal magnetic resonance imaging showed a fluid collection limited to the greater vulvae. Her blood tests showed an inflammatory pattern with increased C-reactive protein 89 g/L and white blood cells at 12620/*µ*L with neutrophils 82%. Fluid cultures of the vulvar tumefaction grew* Escherichia coli* and* Proteus mirabilis* but the tumefaction failed to resolve after appropriate antibiotic therapy. Unfortunately, excisional biopsy of the mass showed metastasis of a high grade urothelial carcinoma.

## 3. Discussion

The entire female genital tract is at risk for metastases occurrence from intragenital and extragenital primaries most commonly gastrointestinal adenocarcinomas [4]. The vulva is the least metastatic site of the female genital tract with the labia majora being the most common location [5]. It is noteworthy to mention that the occurrence of vulvar metastasis represents an end stage disease as it underlines, in general, a widespread incurable disease [6].

The metastatic patterns of urothelial carcinoma of the bladder are not fully elucidated. Limited data underlines a variable metastatic potential that allows this carcinoma to involve any organ by affecting certain genes and proteins, including p53, E-cadherin, Bcl-2, and insulin-like growth factor binding protein 2 gene [2]. Metastasis occurred commonly in more than one organ with 20–45% of patients having at least three metastases [2, 7]. Possible mechanisms for distant metastasis include vascular or lymphatic spread, seeding, and iatrogenicity. Local recurrence is explained by seeding and multicentricity [8]. In the particular case of metastatic spread of the malignant cells to the vulva, it appears that vulvar metastatic disease is associated with vascular involvement of the vulvar tissue [9]. One paper attributed vulvar metastases to vascular space involvement with the exception of primary transitional cell carcinomas [3].

English literature reports very few cases of vulvar metastasis (Table 1). Moreover, urothelial cancers are less common. Dehner investigated 22 cases of secondary tumors to the vulvae and detected two cases of vulvar metastasis from urothelial carcinoma to the labia majora and perineum [3]. One patient presented with hematuria, dysuria, and incidental labial nodule on physical exam. She received vulvovaginectomy with bilateral inguinal lymphadenopathy and died 130 months later without evidence of disease. The other presented a mobile, painless perineal nodule 19 months after diagnosis of transitional cell carcinoma that received pelvic exenteration and died 39 months after recurrence with local and pulmonary metastasis. In one paper, Boardman et al. report two cases of metastatic bladder transitional cell carcinoma to the vulvae. One case presented at diagnosis a synchronous metastatic disease with a pagetoid manifestation. She received radical vulvectomy and systemic chemotherapy and was in complete remission at 8 months. The second case had the same manifestation one year after her initial diagnosis. She was treated with radical vulvectomy and died of disease 65 months later. An interesting series by MD Anderson Cancer Center reports 66 cases of metastatic tumors to the vulva. The mean age at diagnosis was 54.8 years (range: 18 to 84 years) [7]. Most commonly, patients presented with vulvar mass 59%, pain 11%, and ulceration 8%. Whilst only 9% had unknown primary tumor, it was of urothelial origin in 3% of cases and nonurothelial origin in the remainder. In this series, two patients presented with urothelial tumors manifesting by a mass or a cyst. Both patients received radiotherapy and died of disease at 2 and 12 months for the bladder and urethral disease successively.

The core tip of this case is to maintain a high index of suspicion for vulvar metastasis in women with extremely unusual vulvar swellings and a past history of cancer. Early detection and appropriate treatment of these unusual sites could highly affect the prognosis of these patients.

## Figures and Tables

**Table 1:** Review of the literature-clinicopathological features of metastatic urothelial carcinoma to the vulva.

Authors	Number	Age (years)	Symptoms	Primary	Interval	Sites of metastasis	Tissue involvement	Other metastases	Treatment	Follow-up
Dehner (1973) [3]	2	48	Hematuria and disuria	Urothelial urethra	Synchronous	Labia majora	NR	No	Vulvovaginectomy with bilateral inguinal lymphadenopathy	Living well at 130 months
		57	Painless vulvar nodule	Urothelial urethra	19 months	Perineum	NR	No	Pelvic exenteration	Died of disease at 58 months

Mazur et al. (1984) [4]	1	NR	NR	NR	NR	NR	NR	NR	NR	NR

Cohen et al. (1988) [9]	1	NR	Painless nodule	Kidney	>8 months	Vulvae	NR	No	NR	NR

Lerner et al. (1999) [5]	1	78	Painful nodule	Urothelial bladder	Synchronous	Vulvae	NR	No	NR	NR

Boardman et al. (2001) [10]	2	71	Pagetoid vulvae	Urothelial CIS bladder	12 months	Vulvae	NR	Yes	Radical vulvectomy	Died of disease at 65 months
		64	Pagetoid vulvae	Urothelial CIS bladder	Synchronous	Vulvae	NR	Yes	Radical vulvectomy + chemotherapy	Follow-up at 8 months: no disease

Neto et al. (2003) [6]	2	54	Cyst	Bladder	2 months	Labia majora	ep + der + subc	Yes	Xrt	Died of disease at 2 months
		53	Mass	Urethra	12 months	Labia majora	ep + der + subc	Yes	Xrt	Died of disease at 12 months

Our case (2015)	1	63	Painless nodule	Bladder	12 months	Vulvae	NR	Yes		

der: dermis; ep: epidermis; mo: month; NR: not reported, Sepi: squamous epithelium; Subc: subcutis; Xrt: radiotherapy.

## References

[1] Convington E. E., Brendle W. K. (1964). Breast carcinoma with vulvar metastasis. *Obstetrics and Gynecology*.

[2] Shinagare A. B., Ramaiya N. H., Jagannathan J. P., Fennessy F. M., Taplin M.-E., Van Den Abbeele A. D. (2011). Metastatic pattern of bladder cancer: correlation with the characteristics of the primary tumor. *American Journal of Roentgenology*.

[3] Dehner L. P. (1973). Metastatic and secondary tumors of the vulva. *Obstetrics and Gynecology*.

[4] Mazur M. T., Hsueh S., Gersell D. J. (1984). Metastases to the female genital tract: analysis of 325 cases. *Cancer*.

[5] Lerner L. B., Andrews S. J., Gonzalez J. L., Heaney J. A., Currie J. L. (1999). Vulvar metastases secondary to transitional cell carcinoma of the bladder: a case report. *Journal of Reproductive Medicine*.

[6] Neto A. G., Deavers M. T., Silva E. G., Malpica A. (2003). Metastatic tumors of the vulva: a clinicopathologic study of 66 cases. *American Journal of Surgical Pathology*.

[7] Sengeløv L., Kamby C., von der Maase H. (1996). Pattern of metastases in relation to characteristics of primary tumor and treatment in patients with disseminated urothelial carcinoma. *Journal of Urology*.

[8] Guven E. O., Kilciler M., Bedir S., Avci A., Ozgok Y. (2007). Transitional cell carcinoma of the clitoris: direct implantation or metastasis. *International Urology and Nephrology*.

[9] Cohen R., Margolius K. A., Guidozzi F. (1988). Non-gynaecological metastases to the vulva and vagina. *South African Medical Journal*.

[10] Boardman C. H., Webb M. J., Cheville J. C., Lerner S. E., Zincke H. (2001). Transitional cell carcinoma of the bladder mimicking recurrent paget's disease of the vulva: report of two cases, with one occurring in a myocutaneous flap. *Gynecologic Oncology*.

